# Effects of a combined protein and antioxidant supplement on recovery of muscle function and soreness following eccentric exercise

**DOI:** 10.1186/s12970-017-0179-6

**Published:** 2017-07-03

**Authors:** Stephen J. Ives, Samuel Bloom, Alexs Matias, Noelle Morrow, Natalya Martins, Yookee Roh, Daniel Ebenstein, Gabriel O’Brien, Daniela Escudero, Kevin Brito, Leah Glickman, Scott Connelly, Paul J. Arciero

**Affiliations:** 1 Human Nutrition & Metabolism Laboratory, Health and Exercise Sciences Department, Skidmore College, 815 N. Broadway, Saratoga Springs, NY 12866 USA; 2 Scott Connelly Foundation, Corona Del Mar, CA USA

**Keywords:** Protein intake, Antioxidant supplementation, Eccentric exercise, Muscle damage, Free radicals, Muscle function

## Abstract

**Background:**

An acute bout of eccentric contractions (ECC) cause muscle fiber damage, inflammation, impaired muscle function (MF) and muscle soreness (MS). Individually, protein (PRO) and antioxidant (AO) supplementation may improve some aspects of recovery from ECC, though have yet to be combined. We sought to determine if combined PRO and AO supplementation (PRO + AO) improves MS and MF following damaging ECC over PRO alone.

**Methods:**

Sixty sedentary college-aged males participated in a randomized, single–blind, parallel design study of peak isometric torque (PIMT), peak isokinetic torque (PIKT), thigh circumference (TC), and muscle soreness (MS) of knee extensor muscles measured at baseline, immediately after and 1, 2, 6, and 24 h after completion of 100 maximal ECC. Immediately, 6 h, and 22 h post-ECC, participants consumed either: carbohydrate control (CHO; *n* = 14), PRO (*n* = 16), or PRO + AO (*n* = 17).

**Results:**

At baseline MS, TC, MF, macro- and micro-nutrient intakes, and total work during the ECC were not different between groups (*p* > 0.05). PIMT and PIKT (both −25%∆), TC (~1%∆) and MS (~35%∆) all changed with time (*p* < 0.05). We observed a group by time effect for PIKT (PRO + AO and PRO > CHO, *p* < 0.05). At 24 h post ECC, there was a trend towards improved relative PIMT (~11%) and PIKT (~17%) for PRO + AO (~17%) and PRO (~11%) compared to CHO. An interaction indicated PRO + AO had lowest MS over time (PRO + AO > PRO & CHO, *p* < 0.05).

**Conclusions:**

Our results suggest PRO facilitates recovery of muscle function within 24 h following ECC, and addition of AO ameliorates MS more than PRO or CHO alone.

**Electronic supplementary material:**

The online version of this article (doi:10.1186/s12970-017-0179-6) contains supplementary material, which is available to authorized users.

## Background

Many athletic (e.g. CrossFit, wrestling, powerlifting, tennis, etc.) or occupational (e.g. military, firefighting, etc.) endeavors involve high intensity and/or eccentric muscle contractions with repeated bouts of exercise within a 24-h time frame, with as little as 0–6 h between efforts, or on succeeding days. An eccentric contraction occurs when a muscle produces force while lengthening and is used to resist an external force. Eccentric actions, more so than concentric or isometric contractions, can result in significant structural muscle damage and soreness. Initially, eccentric exercise induces damage via mechanical stress [1], which can impair excitation contraction coupling, followed by subsequent “damage” of metabolic origin [2]. Secondarily, in response to mechanical damage, muscle fibers release pro-inflammatory cytokines (IL-1β, TNF-α, IL-6, and IL-8) that attract neutrophils and macrophages into muscle to ultimately repair the damaged tissue [2, 3]. Although not a direct effect of the inflammatory response, muscle soreness following eccentric exercise is due to increased nociceptor and mechanoreceptor sensitivity to tissue breakdown by-products. Within muscle, leukocytes release reactive oxygen species (ROS) while cytokines may activate ROS-generating enzymes [4]. These ROS, while performing necessary breakdown of damaged tissue, may also react with healthy cellular structures, further impairing muscle function [2]. Collectively, the eccentric contraction-induced mechanical and metabolic disturbances in muscle may acutely impair muscle function and performance and increase perceived soreness [1, 2, 5, 6]. Importantly, in certain athletic or occupational settings, repeated bouts of high intensity eccentric exercise with inadequate recovery may be required and thus it’s paramount to develop strategies to enhance recovery in an acute time frame.

Though antioxidant supplementation is unlikely to change the inflammatory cascade [3], several studies have demonstrated the efficacy of antioxidant supplements, particularly those containing anthocyanin-rich berry extracts, in attenuating the acute decline in muscle strength due to eccentric exercise-induced muscle damage [7–9], assumedly through abatement of eccentric-induced ROS. However, the efficacy may be specific to the antioxidant [10–12]. In the current study, we utilized an anthocyanin-rich berry compound (OptiBerry®) with high Oxygen-Radical Absorbing Capacity (ORAC score 43) and proven antioxidant efficacy [13, 14]. While it is debatable whether long term use of antioxidants is advised, as they may hinder the long term adaptation [15], acute supplementation following high intensity resistance exercise (e.g. eccentric contractions) appears to be effective towards restoring muscle function [7–9] and attenuating muscle soreness [16].

Post-exercise supplementation with protein or amino acids may also acutely enhance recovery from eccentric exercise [2, 17–21]. Specifically, ingestion of a whey protein supplement has been shown to enhance recovery of muscle function within 24 h of eccentric exercise in untrained individuals [17]. This enhanced recovery is likely due to elevated levels of free amino acids in the blood leading to increased protein synthesis and attenuated protein breakdown [22]. Additionally, protein supplementation increases satellite cell proliferation following recovery from eccentric exercise [23], a phenomenon important for the recovery of force generation [24]. However, to date, while supplementation with either protein or antioxidants have been demonstrated to be advantageous, no studies have examined the potential synergy of a combined protein (PRO) and antioxidant (AO) supplementation (PRO + AO) on muscle recovery and soreness following eccentric exercise.

Therefore, the purpose of this study was to determine the effects of PRO + AO on muscle soreness and muscle function following fatiguing eccentric contractions. It was hypothesized that the ingestion of recovery doses of PRO + AO following fatiguing eccentric exercise of the knee extensor muscles would reduce perceived muscle soreness and attenuate the decline in muscle function within 24 h more than a carbohydrate control (CHO) or protein (PRO) alone.

## Methods

### Subjects and general procedures

Sixty male college-aged students, aged 18–30, were recruited by public advertisement and word-of-mouth. Participants were relatively sedentary and did not engage in regular physical activity more than twice per week for the purpose of improving or maintaining their physical fitness. Participants had not undertaken resistance training of the quadriceps muscles and had not experienced delayed onset muscle soreness (DOMS) in their quadriceps muscles during the 3 months prior to the start of the study. Participants were free of knee, quadriceps, and other musculoskeletal, or medical problems which could interfere with their ability to perform the required exercise. Participants could not have had a previous allergic or sensitivity response to dairy proteins. All volunteers completed a Health History Questionnaire and physical activity readiness questionnaire (PAR-Q) to assess for eligibility and provided written informed consent. Approval for this study was granted by the Human Subjects Institutional Review Board of Skidmore College (IRB#1503–451) and is in agreement with the Declaration of Helsinki as revised in 1983.

### Study design

**Fig. 1:**
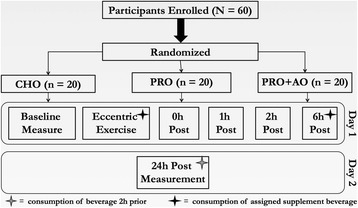
Experimental Overview of the Study

This study was conducted using a randomized, single-blind, placebo-controlled, parallel design (Fig. 1). Subjects were randomly assigned to consume 250 ml of sugar flavored water (CHO control; 31 g of Country Time Berry Lemonade, H.J. Heinz Company Brands LLC. Mendota Heights, MN), 250 ml of water containing 31 g of whey protein hydrolysate (PRO) (Progenex Recovery (Glanbia Thermax 690), Progenex Holdings, LLC, North Salt Lake, UT)) and non-caloric berry flavored powder (Raspberry Lemonade, Crystal Light; Kraft Heinz Company, Glenview IL), or water containing 31 g of whey protein hydrolysate and powdered antioxidants (PRO + AO) (Milk Specialties, MSG 9503, Eden Prairie, MN USA; 100 mg berry extract, OptiBerry®, InterHealth – Lonza, Benicia, CA), all of which were isocaloric (~124 Kcal) and isovolumetric. The anthocyanin-rich berry blend (OptiBerry®) used in this study was a berry powder (80% solubility; standardized to 4 different anthocyanins) extract containing wild blueberry, bilberry, cranberry, elderberry, raspberry seeds, and strawberry per serving and has been demonstrated as a safe and effective antioxidant to inhibit inflammatory markers, and support vascular health [13, 14, 25, 26]. Carbohydrate was chosen as a control as CHO supplementation has not been demonstrated to enhance or impair the recovery from eccentric exercise [27, 28]. Supplements were consumed within a 2 min period after the 100 ECC, and then immediately after the assessments at 6 h, as well as within 2 h prior to the 24 h assessment. Additionally, subjects reported their perceived like/dislike of the supplements via a Hedonic 9-point scale, where the anchor point towards the far left was “Strongest Imaginable Dislike” and to the right was “Strongest Imaginable Like.” Assessment of taste was included as this is often a determining factor in adherence to nutritional supplementation use.

To minimize the impact of diet, all subjects were given an example low antioxidant diet to follow for the day preceding and the day of the study (See Additional file 1: Table S1). To assess dietary intakes between groups and determine compliance a 2 day dietary record was obtained over the study period and analyzed. To characterize the participants, height and weight were measured using standard techniques and body fat percentage, body fat mass, and fat-free mass were measured using the air displacement plethysmography technique (BodPod, Cosmed, Chicago, IL). Volunteers were instructed to refrain from taking over-the-counter medication, “cold & flu” treatments, analgesics, aspirin or other anti-inflammatory preparations for at least 7 days prior to the study, and to abstain from caffeine and alcohol for 24 and 48 h prior to the study, respectively. Subjects reported to the lab after an overnight fast (~12 a.m). As a means of standardization, prior to the 100 maximal ECC and the 24 h assessment, subjects were given a standard breakfast bar (Nature Valley ‘Crunchy Oats ‘n Honey’, General Mills, Inc., Minneapolis, MN) to consume.

### Assessment of muscle function

Assessments of peak isokinetic torque (PIKT) and peak isometric torque (PIMT) of the knee extensor muscles of the right leg, as well as perceived muscle soreness (MS) and thigh circumference measures were obtained prior to the performance of 100 maximal ECCs of the knee extensors of the right leg, immediately after ECC, and at 1, 2, 6, and 24 h post-ECC (Fig. 1). All measurements were obtained from the right leg on all study participants and all non-involved body parts were immobilized throughout all Cybex testing to remove superfluous movement. Both static and dynamic muscle actions were chosen as previous research has suggested the temporal response between muscle actions may differ [6], and dynamic muscle actions more closely mimic those often used in sport or occupational performance. PIKT and PIMT were assessed using the HUMAC NORM (Cybex) ergometer which has been demonstrated to be a reliable ergometer [29, 30]. After 5 min of easy cycling (~1 kp) on an ergometer (Monark 828E; Vansbro, Sweden), participants subsequently performed a warm up set of 3 repetitions on the Cybex. PIMT was determined by using the best of 3 maximal isometric contractions of the knee extensors with the knee flexed at an angle of 90°. For isokinetic measurements, after a warm up set of 5 repetitions, subjects performed 1 set of 5 repetitions of isokinetic contractions of the knee extensors, through an individually determined range of motion (~90°) at an angular velocity of 40°^s-1^. The highest value obtained during the 5 repetitions was recorded and ranged from 120 to 150 ft.·lb. of peak torque. The total warm up lasted approximately 7–10 min. As eccentric contractions may alter sarcomere length, and the optimal length for tension development [1], optimal joint angle, a surrogate for length, was recorded, amongst other variables obtained from the dynamometer. Body position, approximate axis of rotation of the knee joint and dynamometer lever arm length were recorded and thus consistent for all trials for each volunteer. Verbal encouragement was provided during all contractions to ensure maximal effort and was consistent between sessions and participants. The minimum eccentric torque needed to lower the arm and initiate the dynamometer was ~5 ft lb.

### Muscle soreness

Muscle soreness was evaluated using a visual analog scale (VAS) [31]. The VAS consisted of a 100 mm long horizontal line with anchor points on either side stating “no soreness” on the far left and “worst soreness possible” on the far right. Subjects were seated with right leg in passive 90° of flexion at the beginning of the soreness evaluation and instructed to extend the knee of the right leg (parallel to the floor) to full extension. Subjects then placed a mark at the point on the VAS corresponding to their perception of the soreness of the right quadriceps muscle. This was done both unweighted and weighted with a 5-kg mass suspended from the ankle. Muscle soreness was quantified by using the measured distance (mm) from the left end of the continuum to the mark made by the subject. Additionally, a Gulick tape measure was used to quantify the circumference of the right thigh at the midpoint between the greater trochanter of the femur and the knee joint to assess for localized swelling. This location was marked (with a permanent marker) as means of standardization and to maintain consistency during follow-up measurements.

### Eccentric exercise

On the first day of testing, following baseline measures, subjects performed 100 maximal eccentric contractions (ECCs) of the knee extensors of the right leg on a HUMAC NORM isokinetic dynamometer (Boston, USA) at an angular velocity of 40°^s-1^ through an 80° range of motion (Fig. 1). To the initiate each ECC, subjects performed a brief concentric contraction against the dynamometer lever arm of approximately 5 ft lb., and then with continual maximal effort resisted the force of the ergometer lengthening their knee extensors. The knee extensors were relaxed at the end of each ECC, and during the recovery phase the relaxed leg was returned to the starting position by the dynamometer. The dynamometer provides real-time feedback to monitor effort during the ECC. This protocol was chosen as it has been used previously, with success, to determine the impact of dietary interventions on muscle soreness and function [17]. Verbal encouragement was provided to ensure maximal effort in a consistent manner during all contractions for all participants. Those who didn’t exhibit a ≥ 10% decline in peak isometric torque at the 0 h measure, immediately after the ECC, were excluded from the study.

### Data analysis

A one way analysis of variance (ANOVA) was used to compare baseline parameters between the three supplement groups. To determine the effects of the supplements (control vs PRO vs PRO + AO) on the dependent measures (PIMT, VAS, and thigh circumference) over time, two-way mixed model ANOVA (group (3) x time (6)) were used. Where a significant main effect was observed, a post hoc analysis was conducted to identify differences between means using Tukey’s HSD test. To account for potential minor variations in baseline muscle function (ie. PIMT, PIKT), muscle function was normalized to initial and represented as the change from baseline. Alpha was set, a priori, at 0.05 for all comparisons. All data was presented as mean ± standard error of the mean (SE).

## Results

### Participant characteristics

No significant differences were observed for age, height, weight, percent body fat, fat free mass, and initial thigh circumference between groups (Table 1). Due to failure to meet the ≥10% decline in peak isometric torque following ECC, study completion rates were 70% (14/20), 80% (16/20), and 85% (17/20) for CHO, PRO, and PRO + AO groups, respectively. There were no significant differences between the groups at the start of ECC testing.

**Table 1:** Subject characteristics by group

Variable	CHO (*n* = 14)	PRO (*n* = 16)	PRO + AO (*n* = 17)	*p*-value
Age (yrs)	21.3 ± 1.0	20.9 ± 0.5	20.6 ± 0.3	0.76
Height (cm)	175.4 ± 1.9	177.2 ± 1.8	180.5 ± 1.2	0.09
Weight (kg)	71.8 ± 4.1	72.5 ± 3.6	72.2 ± 2.6	0.99
Body Fat (%)	17.0 ± 2.6	14.0 ± 1.7	14.6 ± 1.2	0.56
Fat Free Mass (kg)	74.9 ± 9.8	62.8 ± 2.6	61.2 ± 1.8	0.18
Thigh Circumference (cm)	53.5 ± 2.1	53.1 ± 1.5	54.3 ± 1.2	0.85

Data are mean ± SE

### Dietary record and taste analysis

There was no significant difference (*p* > 0.05) in macronutrient dietary consumption (kilocalories, protein, fat, carbohydrate) for all three treatment groups for the day prior and day of the initial testing, as assessed using 2 day food logs (Table 2). There were no differences in vitamin A, vitamin C, potassium, sodium, calcium, and iron intake between groups (Table 2). A significant difference was observed in cholesterol intake for the day prior to initial testing where CHO group had greater intake than PRO and PRO + AO (*p* = 0.03) (Table 2).

**Table 2:** Dietary analysis from 2 day food log, not including study supplementation

Variable		CHO (*n =* 14)	PRO (*n* = 16)	PRO + AO (*n* = 17)	*p*-value
Calories (kcal)	Day 1	1917 ± 138	2069 ± 229	1861 ± 182	0.42
	Day 2	1655 ± 137	1725 ± 210	1626 ± 151	0.81
Carbohydrates (g)	Day 1	198 ± 11 (41%)	253 ± 40 (49%)	217 ± 26 (47%)	0.74
	Day 2	190 ± 27 (46%)	209 ± 40 (48%)	179 ± 21 (44%)	0.59
Fat (g)	Day 1	80 ± 10 (37%)	74 ± 8 (32%)	68 ± 9 (33%)	0.35
	Day 2	67 ± 6 (36%)	55 ± 10 (29%)	55 ± 9 (30%)	0.71
Protein (g)	Day 1	93 ± 7 (19%)	98 ± 9 (19%)	88 ± 6 (19%)	0.23
	Day 2	98 ± 11 (24%)	94 ± 14 (22%)	81 ± 8 (20%)	0.54
Protein (g/kg)	Day 1	1.35 ± 0.23	1.40 ± 0.24	1.13 ± 0.12	0.29
	Day 2	1.40 ± 0.21	1.14 ± 0.20	1.11 ± 0.13	0.57
Vitamin A (mg)	Day 1	152 ± 70	64 ± 16	65 ± 20	0.20
	Day 2	48 ± 18	44 ± 13	104 ± 35	0.23
Vitamin C (mg)	Day 1	171 ± 59	181 ± 62	79 ± 27	0.26
	Day 2	72 ± 19	34 ± 16	74 ± 19	0.29
Sodium (mg)	Day 1	2458 ± 129	2667 ± 353	3068 ± 622	0.73
	Day 2	2439 ± 414	1781 ± 330	1995 ± 436	0.36
Potassium (mg)	Day 1	1113 ± 286	1103 ± 144	1229 ± 320	0.79
	Day 2	1549 ± 300	1303 ± 238	1322 ± 260	0.68
Calcium (mg)	Day 1	84 ± 28	67 ± 18	51 ± 7	0.43
	Day 2	54 ± 9	46 ± 10	44 ± 11	0.84
Iron (mg)	Day 1	70 ± 23	74 ± 18	59 ± 10	0.78
	Day 2	55 ± 7	41 ± 6	53 ± 8	0.30
Cholesterol (mg)	Day 1	582 ± 134	291 ± 61	250 ± 70	0.03
	Day 2	344 ± 86	414 ± 226	280 ± 94	0.79

Data are mean ± SE

Regarding taste of the supplements, CHO had a significantly greater average score for taste on the Hedonic scale than the PRO supplement (*p* < 0.001), however, there was no difference in taste score between PRO + AO and CHO (*p* = 0.079) or PRO + AO and PRO (*p* = 0.111).

### Muscle function

At baseline, there was no significant differences in both isometric (*p* = 0.529) and isokinetic (*p* = 0.575) muscle function between all three treatment groups (Table 3). During the ECC, total eccentric work (CHO: 13,639 ± 1036 ft·lbs, PRO: 12,458 ± 622 ft·lbs, PRO + AO: 13,518 ± 431 ft·lbs) and peak eccentric torque (CHO: 196 ± 13 ft·lbs, PRO: 178 ± 8 ft·lbs, PRO + AO: 206 ± 8 ft·lbs) during the eccentric exercise were not significantly different between groups.

**Table 3:** Baseline measures of isometric and isokinetic muscle function

Variable	CHO (*n* = 14)	PRO (*n* = 16)	PRO + AO (*n* = 17)	*p*-value
Isometric				
Peak Torque (ft·lb)	152 ± 12.3	159 ± 8.8	167 ± 7.2	0.59
Average Torque (ft·lb)	134 ± 11.2	139 ± 7.7	147 ± 6.8	0.56
Slope (ft·lb./s)	138 ± 15.4	122 ± 19.1	134 ± 21.3	0.84
Time to ½ Peak (sec)	0.16 ± 0.02	0.24 ± 0.03	0.19 ± 0.04	0.22
Time to Peak (sec)	1.94 ± 0.29	2.38 ± 0.30	2.56 ± 2.46	0.30
Isokinetic				
Peak Torque (ft·lb)	138 ± 9.9	126 ± 7.9	135 ± 7.1	0.58
Work per Rep (*J*)	133 ± 10.6	124 ± 8.3	127 ± 8.9	0.23
Average Power (W)	78 ± 6.6	70 ± 5.2	74 ± 4.9	0.46
Joint Angle at Peak (deg)	66 ± 2.5	68 ± 2.7	69 ± 2.2	0.69
Time to Peak (sec)	0.85 ± 0.09	0.84 ± 0.07	0.73 ± 0.08	0.48
Time Peak Held (sec)	0.04 ± 0.01	0.09 ± 0.05	0.07 ± 0.01	0.60
Force Decay Time (sec)	1.43 ± 0.05	1.48 ± 0.06	1.42 ± 0.08	0.79
Reciprocal Delay (sec)	0.65 ± 0.21	0.34 ± 0.08	0.36 ± 0.10	0.24
Delay Time (sec)	−0.07 ± 0.08	−0.04 ± 0.00	−0.05 ± 0.01	0.89

Data are mean ± SE

**Fig. 2:**
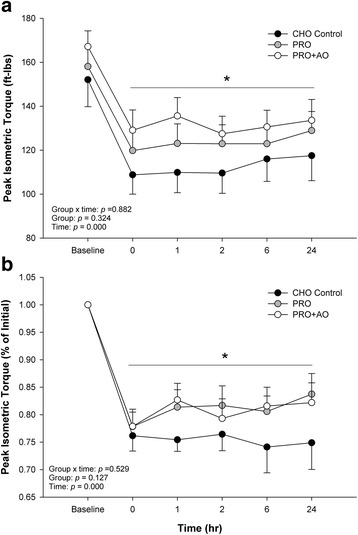
The Impact of Supplementation (CHO, PRO, PRO + AO) on Absolute Isometric Muscle Function (Panel **a**) and Change in Isometric Muscle Function from Baseline (Panel **b**). * *p* < 0.05 individual time points vs. baseline. Data are mean ± SE

Peak isometric torque, expectedly, was significantly reduced (*p* < 0.001) following the ECC (Fig. 2a). However, there was no main effect of group (*p* = 0.324) nor an interaction of group over time (*p* = 0.882) for PIMT. When expressed as percent of baseline (Fig. 2b) there was again an effect of time (~25% decrease, *p* < 0.001), but no significant group effect (*p* = 0.127) or group over time interaction effect (*p* = 0.529) for PIMT.

**Fig. 3:**
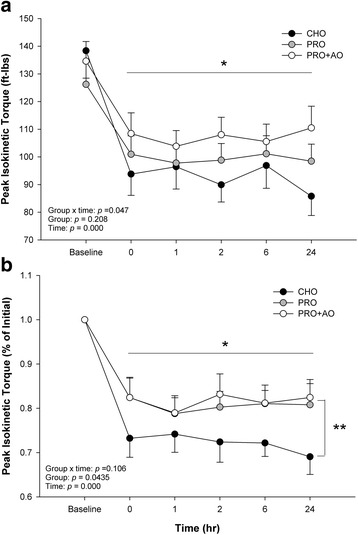
The Impact of Supplementation (CHO, PRO, PRO + AO) on Absolute Isokinetic Muscle Function (Panel **a**) and Change in Isokinetic Muscle Function from Baseline (Panel **b**). * *p* < 0.05 individual time points vs. baseline; ** *p* < 0.05 CHO vs. PRO and PRO + AO. Data are mean ± SE

Peak isokinetic torque, also exhibited a significant drop in torque over time (Fig. 3a) (*p* < 0.000), with a significant interaction effect for PIKT for group over time (*p* = 0.047, Fig. 3a), where the PRO and PRO + AO groups had greater forces than the CHO group. When post hoc tests were conducted, a significant difference was observed between CHO group and PRO and PRO + AO groups at the 24 h time point (*p* = 0.026, Fig. 3a), though, the main effect for group was not significant (*p* = 0.208). When expressed as percent of baseline (Fig. 3b), the effect of time was again significant (~25% decline, *p* < 0.001), and there was a significant main effect for group (*p* = 0.0435) but no interaction effect of group over time (*p* = 0.106) for PIKT (Fig. 3b). Post hoc testing of group effects revealed that both PRO (11% difference) and PRO + AO (17% difference) groups had significantly higher, on average, peak isokinetic torques than the CHO control group.

### Muscle soreness and thigh circumference

**Fig. 4:**
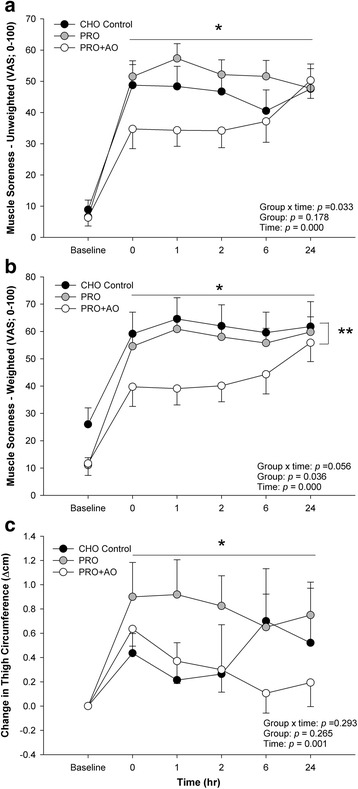
The Impact of Supplementation on Changes in Muscle Soreness and Swelling. **a** Unweighted Visual analog scale for soreness, **b** Weighted VAS for soreness, and **c** Change in Thigh Circumference (CHO *n* = 14, PRO *n* = 16, PRO + AO *n* = 17). *p < 0.05 individual time points vs. baseline; ** *p* = 0.027, CHO vs. PRO + AO. Data are mean ± SE

All groups experienced a significant increase in unweighted and weighted muscle soreness following the ECC (Fig. 4a–b, *p* < 0.001). For unweighted VAS, there was a significant interaction of group and time (*p* = 0.033), where the PRO + AO group had the lowest soreness over time. Post hoc testing showed PRO + AO was lower than PRO at 0, 1, 2, and 6 h post ECC (*p* = 0.02, 0.001, 0.009, and 0.04), and tended to be lower than CHO alone at 0, 1, and 2 (*p* = 0.07, 0.05, and 0.07). However, there was no significant main effect of group (*p* = 0.178). For the weighted VAS, there was a trend for an interaction effect of group over time (*p* = 0.056) and a significant group effect (*p* = 0.036, Fig. 4b), with PRO + AO reporting the lowest soreness compared to PRO and CHO. Finally, there was a significant increase in thigh circumference over time (*p* = 0.001, Fig. 4c), but no significant group (*p* = 0.265) or group by time interaction effects (*p* = 0.293).

## Discussion

The purpose of this study was to determine if a combined protein and antioxidant supplement (PRO + AO) was more effective than either protein (PRO) or carbohydrate control (CHO) alone in restoring muscle soreness and muscle function following fatiguing eccentric contractions (ECC) during the acute 24 h phase. The primary findings of this study demonstrate that during the acute 24 h period following fatiguing ECC exercise: 1) muscle function is significantly reduced and muscle soreness and thigh circumference are increased; 2) both groups that supplemented with PRO tended to have better isometric muscle function, and significantly greater isokinetic muscle function over CHO control; and 3) combined protein and antioxidant supplementation (PRO + AO) tended to have better absolute isokinetic torque and significantly less perceived soreness over time when compared to protein alone (PRO) or a carbohydrate (CHO) control. Collectively, the current findings support protein supplementation to enhance recovery of muscle function and the addition of antioxidants to act synergistically to reduce perceived muscle soreness, in the hours immediately following ECC exercise. Thus, the combination of protein and antioxidants may be an important consideration in developing and implementing strategies to aid in muscle recovery during the acute (0-24 h) time period following a bout of exhaustive eccentric exercise.

### Muscle function

Indeed many athletic events that involve high intensity and/or eccentric contractions often require short turnaround times to subsequent events (0–6 h), which does not allow for adequate muscle recovery. For instance, the CrossFit games, wrestling tournaments, powerlifting competitions, tennis tournaments, etc., all require multiple high intensity bouts within a given day, and perhaps on successive days. Thus, developing supplementation strategies to improve recovery and reduce muscle soreness, might improve performance in such settings. In terms of aiding recovery of muscle function from resistance exercise or eccentric exercise, supplementation with protein or amino acids post-exercise has been demonstrated to enhance recovery [17, 19, 20, 22]. Regarding isometric muscle function, previous work by Buckley and colleagues [17] found that consumption of whey protein hydrolysate resulted in a quicker recovery in peak isometric torque following ECC, with complete recovery at 6 h post exercise. Similarly, the current study found at 24 h post ECC, peak isometric torque was higher, on average, though not statistically, in both PRO + AO and PRO groups compared to the CHO group, by 16 and 11 ft lb., respectively (Fig. 2a), or approximately a 38% difference in peak isometric torque (% of initial) between protein supplemented groups and CHO control (Fig. 2b). However, unlike the Buckley et al. study [17], and others [18], which found supplementation with protein completely restored and even surpassed baseline peak isometric torque [17], we did not find resolution of force within the same observed timeframe (Fig. 2), though the PRO supplemented groups seemed to have a positive slope towards recovery, where the CHO did not. Although total work between groups was not reported in these earlier studies, even concentric muscle force contractions are suppressed following high intensity work [32]. In any case and even though our measurements did not extend to 48 h post ECC, the pattern of our reported muscle function data (Figs. 2 and 3) seems to suggest we observed a similar pattern as others in this regard [23, 28, 33].

As muscle function, broadly defined, is impaired following ECC, the magnitude and temporal pattern may depend upon contraction type; specifically, static versus dynamic, and concentric versus eccentric [5, 6]. Thus, it’s paramount to examine both static and dynamic muscle function, with the latter likely being more important for performance. Examination of dynamic peak isokinetic torque from the current study, we found a group by time interaction when expressed as absolute torque, whereby CHO control experiences the most dramatic decline in torque, followed by PRO, whereas PRO + AO appears least affected (Fig. 3a). Similarly, when expressed as percent of initial peak torque, there was a group effect with PRO and PRO + AO groups having a higher on average peak isokinetic torque (Fig. 3b), or approximately a 17% difference between PRO supplemented groups and CHO control group (Fig. 3b). Thus, in agreement with previous studies [18], the present study suggests protein supplementation following ECC does, in fact, improve recovery of dynamic muscle function. However, it is unlikely that dynamic muscle function returns to baseline levels within an acute time frame (<48 h), independent of supplementation [23, 28, 33].

Furthermore, previous research has demonstrated that supplementation with antioxidants derived from fruit improves recovery of muscle function following fatiguing eccentric exercise [7–9], though not all agree [34]. These studies contained either whole fruit or pasteurized fruit juices containing a combination of anthocyanins with phenolic compounds, flavonoids, ellagitannins, and/or ellagic acid. In the current study, using an antioxidant derived from anthocyanin extract from mixed berries, we found no synergistic effect of supplementing with both protein and antioxidants, or the antioxidant offered no obvious additional benefit beyond that of protein alone (Figs. 2 and 3). In agreement, previous studies have also found no effect of antioxidant supplementation (using either vitamin C or fruit, berry and vegetable concentrate) on muscle recovery [10, 11, 34]. In contrast, previous studies using either a berry or fruit derived antioxidant supplement demonstrate an interaction between groups with no clear direction of improved muscle function [9] or show enhanced recovery of muscle function with a pre-loading period prior to ECC [8]. Collectively, future work is needed to determine if optimal dosing strategy, types of antioxidants, may influence the impact of antioxidant supplementation on muscle function following fatiguing eccentric exercise.

### Muscle soreness

Decline in muscle function, athletic performance, and intensification of muscle soreness are all correlated to the damaging effects of eccentric exercise on muscle. Studies investigating the effect of protein or amino acids on muscle soreness following ECC have yielded both positive [19, 20, 22] and null [17, 18, 23, 28] effects. In the present study, using both unweighted and weighted conditions to assess perceived muscle soreness, we show only protein and antioxidant supplementation (PRO + AO) favorably impacts perceived muscle soreness (via visual analog scales) for both weighted and unweighted conditions (Fig. 4a and b). While there was a significant time effect for increased thigh circumference indicative of swelling and localized inflammatory responses, no group or group by time effects were found.

Our finding of significantly lower perceived muscle soreness over time in combined protein and antioxidant (PRO + AO) supplementation compared to PRO and CHO groups is intriguing (Fig. 4a). The current finding supports prior work suggesting antioxidant supplementation may reduce muscle soreness following ECC [7, 11, 34], although the positive effect of antioxidants on muscle soreness is not unanimous [8–10]. Although the precise mechanism(s) for reduced muscle soreness is unknown, it may be related to either the high leucine or essential amino acid content within whey protein [35], antioxidants potential to reduce muscle soreness following ECC exercise [36], or may be related to the many bioactives present within various forms of whey protein. In any case, this finding may prove beneficial in scenarios where subsequent bouts with inadequate recovery are necessitated (e.g. tournament play, multiple sport events, military or occupational scenarios).

### Experimental considerations

While the findings of this study demonstrate improved muscle function, and attenuated muscle soreness with combined protein and antioxidant supplementation, further evaluation of this recovery strategy is necessary. Specifically, the type, dose, and dosing strategy (pre-loading, frequency of dosing, etc.) likely needs optimization. Additionally, while muscle function is paramount, analysis of blood markers of muscle damage and oxidative stress, could provide insight into the underlying mechanisms of protein and antioxidant supplementation in the recovery of muscle soreness and function following eccentric exercise. On the contrary, previous studies which have measured blood markers of oxidative stress or muscle damage (e.g. creatine kinase) that were discordant with function [3, 8, 9, 11, 34], in that muscle function would be improved but not oxidative markers, or vice versa, calling into question the value of such measurements. Additionally, observing the effect of the supplement across a larger time period (up to 92 h or resolution of soreness/function), as well as in trained, competing athletes in which this is primarily intended to benefit, are suggested.

## Conclusions

The current study adds to the growing body of evidence supporting the benefits of ingesting protein following high intensity eccentric exercise and extends it by demonstrating that a combined PRO + AO supplement may further mitigate the eccentric-induced decrements in muscle function and reduce muscle soreness in the acute time frame (0–24 h) following the eccentric bout. Collectively, a combined protein antioxidant supplement is likely beneficial in the recovery from eccentric exercise, and may be relevant for novice exercisers or in athletic or occupational scenarios where a repeated bout is required before full recovery is allowed.

## Additional file

Additional file 1: Table S1. Meal Plan for the day before, day of testing, and following morning before 24 h testing period. (DOCX 12 kb)

## Abbreviations

CHO: Carbohydrate
ECC: Eccentric contractions
MF: Muscle function
MS: Muscle soreness
PIKT: Peak isokinetic torque
PIMT: Peak isometric torque
PRO + AO: Protein and antioxidant
PRO: Protein
TC: Thigh circumference
