# Supplementary material for: Effects of a combined protein and antioxidant supplement on recovery of muscle function and soreness following eccentric exercise
Source: J Int Soc Sports Nutr. 2017 Jul 3;14:21. doi: 10.1186/s12970-017-0179-6 (PMC5496333; doi:10.1186/s12970-017-0179-6)
Supplement: Additional file 1: Table S1. — Meal Plan for the day before, day of testing, and following morning before 24 h testing period. (DOCX 12 kb) [file 12970_2017_179_MOESM1_ESM.docx]

| **Breakfast** | **Lunch** | **Dinner** | **Snacks** |
| --- | --- | --- | --- |
| - Toast, Whole Wheat (2 Slices) - Butter (1 tsp) - Hard Boiled Eggs (2) - White grapes (1) - Water (8 oz) | - Chicken (4 oz) - Rice (1 cup) - Green beans (1 cup) - Banana (1) - Water (8 oz) | - Turkey Burger (4 oz) w/ Whole Wheat Bun & Cheese Slice (1) - Lettuce (1 cup) - Baked potato (1) - Butter (1 tsp) - Water (8 oz) | - Greek Yogurt, Vanilla (1 cup) - Peanut Butter (2 tbsp) - Celery (2 stalks) - Milk (8 oz) |

**Table** S1. Meal Plan for the day before, day of testing, and following morning before 24 hour testing period.
